# Parametric Random Vibration Analysis of an Axially Moving Laminated Shape Memory Alloy Beam Based on Monte Carlo Simulation

**DOI:** 10.3390/ma15020562

**Published:** 2022-01-12

**Authors:** Ying Hao, Ming Gao, Jiajie Gong

**Affiliations:** 1 College of Civil Engineering & Mechanics, Hebei Provincial Key Laboratory of Mechanical Reliability for Heavy Equipments and Large Structures, Yanshan University, Qinhuangdao 066004, China; hy0505@ysu.edu.cn (Y.H.); gongjjss@shu.edu.cn (J.G.); 2 Mechanical and Electronic Engineering College, Shandong Agricultural Equipment Intelligent Engineering Laboratory, Shandong Provincial Key Laboratory of Horticultural Machineries and Equipments, Shandong Agricultural University, Tai’an 271018, China

**Keywords:** axial motion with variable velocity, laminated SMA beam, random parametric excitation, Monte Carlo simulation

## Abstract

The study of the bifurcation, random vibration, chaotic dynamics, and control of laminated composite beams are research hotspots. In this paper, the parametric random vibration of an axially moving laminated shape memory alloy (SMA) beam was investigated. In light of the Timoshenko beam theory and taking into consideration axial motion effects and axial forces, a random dynamic equation of laminated SMA beams was deduced. The Falk’s polynomial constitutive model of SMA was used to simulate the nonlinear random dynamic behavior of the laminated beam. Additionally, the numerical of the probability density function and power spectral density curves was obtained through the Monte Carlo simulation. The results indicated that the large amplitude vibration character of the beam can be caused by random perturbation on axial velocity.

## 1. Introduction

Axially moving structures are widely found in aerospace, civil engineering, machinery, and transportation industries. In theoretical analysis, these structures are modeled as beams, chords, or plates. So far, the nonlinear vibration behaviors of axially moving beams, chords, and plates have been extensively studied. Chen et al. [1] applied a multiple-dimension Lindsted–Poincare (L–P) process to examine internal resonance in the vibration of axially moving beams. Ding et al. [2] used a multi-scale procedure to analyze the stability and steady-state response of axially moving viscoelastic beams. Yang et al. [3] established a dynamics model of an axially moving viscoelastic beam and discussed how axial motion parameters and system viscoelastic coefficients affect the bifurcation behavior of this beam. Liu et al. [4] compared the vibration characteristics of three typical types of axially moving structures—Euler beams, panels, and plates with two opposite sides simply supported and the other two left free. Tang et al. [5] probed into the transverse nonlinear vibration of a viscoelastic plate moving axially with variable velocity. Al-Bedoor et al. [6] found the approximate analytical solution of beam vibration during axial motion. Chen et al. [7] investigated the nonlinear vibration of axially moving beams using the harmonic balance method. Burak et al. [8] used a method of multiple time scales (a perturbation method) to examine the nonlinear vibration and stability of axially moving beams with variable velocity and axial force values. Lenci et al. [9] investigated the nonlinear free oscillations of a planar Timoshenko straight beam using the asymptotic expansion method. Xiao et al. [10], Liu et al. [11], and Jin et al. [12] studied vibration complex problems in other important engineering structures.

Shape memory alloys (SMAs) feature particular shape memory and pseudo elasticity characteristics. They are more sensitive to stress and temperature and are more deformable and elastic than common metals [13]. Laminated beams are a usual form of laminated structures, which are structures composed of SMAs on the surface and linear elastic material in between. Ren et al. [14,15,16] carried out a succession of studies on SMA fiber hybrid laminated beams. They developed a theoretical analysis model describing the natural frequency of a kind of hybrid laminated beam composed of SMA fibers laminated with common fibers and observed the effects of the content, installation angel, and transverse shear deformation of SMA fibers. Collet et al. [17] considered a hypothesis that SMA remains symmetric under tensile, compressive, and temperature loading and tested the dynamical behaviors of an SMA beam by applying external moving loads on the material. Through hierarchical Rayleigh–Ritz simulation, de Matos Junior et al. [18] studied the nonlinearity of the aeroelastic behavior of stiffened SMA hybrid composite (SMAHC) cylindrical plates on a carbon fiber–SMAHC laminated plate. Zhang et al. presented an experimental study on the random vibration of aviation conduits with SMA joints [19]. Razavilar et al. [20] developed a semi-analytical procedure for studying the free vibration and forced vibration of an SMA beam with pseudoelastic behavior. They established the control dynamics equations of a deformation–strain-coupled SMA beam and analyzed its thermodynamic properties with phase trajectory. Nassiri-monfared et al. [21] characterized the thermomechanical behavior of a beam reinforced with SMA elements on an improved Brinson polynomial constitutive model. Zhang [22] tested how external excitation and structural parameters (parameters related to the thickness ratio between the SMA layer and the beam substrate) affect the one-third subharmonic and third superharmonic resonance of an SMA-laminated beam supported at both ends. Nejati et al. [23] analyzed the thermal vibration of SMA hybrid composite double-curved sandwich panels. Samadpour et al. [24] looked into the nonlinear aero-thermal flutter postponement of supersonic-laminated composite beams with SMA.

Given the possibility of complex nonlinear dynamics in the system, such as sharp vibration, the resonance of parametric vibration with forced vibration, bifurcation, and chaos, in response to axial velocity and external excitation, studying the transverse vibration mechanism of axially moving beams is both theoretically and practically useful for optimizing engineering system components. By considering the effects of shear modulus and moment of inertia, Li et al. [25] used the multiple time scale method to examine the steady-state response of an axially moving viscoelastic Timoshenko beam to forced transverse nonlinear vibration. Ding et al. [26] introduced finite support stiffness and investigated the chaotic nonlinear dynamics of an axially moving viscoelastic beam subject to a combination of external excitation and parametric excitation. Ding et al. [27] applied Timoshenko beam theory to the nonlinear dynamics studies of a structure moving axially with high speed for the first time. They derived the static balance equation of the beam, deduced the critical velocity of an axially moving Timoshenko beam and discussed the effects of system parameters on equilibrium bifurcation and critical speed. Wang et al. [28,29] discussed the magneto-elastic primary and internal resonances of axially moving conductive beams in a magnetic field. Tang et al. [30] established the nonlinear dynamics model of an axially accelerating viscoelastic beam by considering the non-uniform boundary conditions induced by the Kelvin viscoelastic constitutive relation. Through a numerical example, they analyzed the effects of material viscoelastic coefficient and axial velocity fluctuation amplitude on steady-state vibration response. Based on the Galerkin method and fourth-order Runge–Kutta method, Shao et al. [31] studied the nonlinear vibration of a thin film moving axially with variable velocity and analyzed its chaotic and bifurcation behaviors in response to changed average velocity and velocity fluctuation amplitude. Sahoo et al. [32] examined the steady-state response of an axially accelerating viscoelastic beam to dual-frequency parametric excitation both analytically and numerically. Through phase diagram, time history, and Poincare mapping analyses, they discovered the Hopf bifurcation, saddle node bifurcation, and pitch-fork bifurcation present in the system. Yang et al. [33] analyzed the stability of a compressible laminated beam moving with variable velocity. In addition, on the problem of random vibration, Hu [34,35,36] analyzed the response and control for random time-delay systems under wide-band random excitations and Harmonic and Wide-Band Noises.

At present, the research on the axially moving continuum mainly focuses on homogeneous materials, and the laminated structure is rarely considered in the modeling process and analysis of axially moving beams. Most of the existing literature considers nonlinear dynamic problems such as the internal resonance, principal resonance, and bifurcation of laminated beams, but there is a lack of research that considers the random disturbance of axial velocity on the nonlinear stochastic dynamic behaviors. In this dissertation, based on the force balance conditions, deformation compatibility equation, and Falk’s polynomial constitutive model of SMA, the random vibration differential equation of laminated SMA beams is derived and numerically solved. The effects of random perturbation intensity and axial velocity on steady-state response are analyzed. 

## 2. Random Vibration Equation of Laminated SMA Beams Moving Axially with Variable Velocity

### 2.1. Polynomial Constitutive Relation of SMA

For an SMA-laminated beam with a complex structure and complicated stress conditions, it is sometimes difficult to obtain the dynamic equation of the system by using other constitutive models, and the nonlinear dynamics characteristics of the system can be easily obtained and analyzed by using this constitutive model. Paiva and Savi’s [34] research shows that the polynomial model can qualitatively describe the dynamic behavior of SMA. This dissertation used this quantic polynomial stress–strain constitutive relation of SMA in Ref. [37], which was written as

(1) $$\sigma =\;{a(T\;-\;T}_{M})\varepsilon \;-\;b\varepsilon^{3}+e\varepsilon^{5}$$

where *a*, *b* and $\;e=\frac{b^{2}}{{4a(T}_{A}{-T}_{M})}$ are material constants; $T_{A}$ is the temperature above which austenite is stable, and $T_{M}$ is the temperature below which marten site is stable;${\;a=1\;\times \;10}^{3}$ MPa/K, ${b=40\;\times \;10}^{6\;}$ MPa/K, ${\;T}_{A}=$313 K, and ${\;T}_{A}=$287 K, which were obtained from experiments in Ref. [37], and the stress–strain curve is shown in Figure 4-2 (Ref. [37]).

### 2.2. Dynamics Equation of Laminated SMA Beams

**Figure 1:**
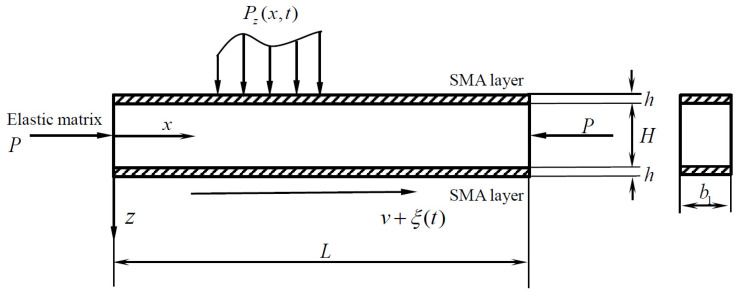
The structural diagram of the axially moving laminated SMA beam.

The geometric model of a laminated beam with length *L*, width $b_{1}$, substrate beam height *H*, and SMA height *h* for both the upper and lower layers is established, as shown in Figure 1. ξ(t) is the random perturbation term on axial velocity, which can be nominally seen as Gaussian white noise with noise intensity *D*. *P_z_*(*x*,*t*) and *P* are the uniform loading and axial pressure acting on the laminated beam.

In the figure, *oxz* is the stationary coordinate system; the axial velocity is  V=v+ξ(t), with traverse displacement being recorded as *ω*(*x*,*t*); the beam transverse velocity is $\frac{dw}{dt}=\frac{\;\partial w}{\partial t}+V\frac{\partial w}{\partial x}$, and the acceleration is $\frac{d^{2}w}{dt^{2}}=\frac{\partial^{2}w}{\partial t^{2}}+2V\frac{\partial^{2}w}{\partial x\partial t}{+V}^{2}\frac{\partial^{2}w}{\partial x^{2}}$. Assuming the forced excitation, *P_z_*(*x*,*t*) = $\;f_{0}$ sin(*γt*) where *f*_0_ is the excitation amplitude. Additionally, the density of the matrix beam is *ρ*, the damping coefficient per unit length is *c*, and the elastic modulus is *E*.

Hao et al. [38,39] gives the force diagram of the micro-body (Figure 2 in Ref. [38]). Introducing the random perturbation of the axial velocity, the random transverse vibration equation of the main beam can be obtained:

(2) $$\begin{array}{c} -E\frac{b_{1}H^{3}}{12}\frac{\partial^{4}w}{\partial x^{4}}+\frac{H}{2}{Eb}_{1}H[{\left(\frac{\partial^{2}w}{\partial x^{2}}\right)}^{2}+\frac{\partial w}{\partial x}\frac{\partial^{3}w}{\partial x^{3}}]{-b}_{1}Hh[a\left({T-T}_{M}\right)\frac{H+h}{2}\frac{\partial^{4}w}{\partial x^{4}}-\frac{3}{8}b{\left(H+h\right)}^{3}{\left(\frac{\partial w}{\partial x}\right)}^{2}\frac{\partial^{4}w}{\partial x^{4}}\\ -\frac{3}{4}b{\left(H+h\right)}^{3}\frac{\partial^{2}w}{\partial x^{2}}{\left(\frac{\partial^{3}w}{\partial x^{3}}\right)}^{2}+\frac{5}{32}e{\left(H+h\right)}^{5}{\left(\frac{\partial^{2}w}{\partial x^{2}}\right)}^{4}\frac{\partial^{4}w}{\partial x^{4}}+\frac{5}{8}e{\left(H+h\right)}^{5}{\left(\frac{\partial^{2}w}{\partial x^{2}}\right)}^{3}{\left(\frac{\partial^{3}w}{\partial x^{3}}\right)}^{2}]\\ -P\frac{\partial^{2}w}{\partial x^{2}}{-\rho b}_{1}H\frac{d^{2}w}{{\mathrm{dt}}^{2}}-c\frac{\partial w}{\partial t}{+P}_{z}=0 \end{array}$$

Considering the first-order mode, the boundary condition of simple support at both ends, the displacement solution can be set as:

(3) $$w(x,t)=f(t)\sin(\frac{\pi }{L}x)$$

If the displacement solution in Equation (3) is introduced to Equation (2), we can obtain:

(4) $$\begin{array}{c} \frac{b_{1}Hh}{2}\left[a\left({T-T}_{M}\right)\left(H+h\right){\left(\frac{\pi }{L}\right)}^{4}f\left(t\right)\sin\left(\frac{\pi }{L}x\right)+\frac{3}{2}b{\left(H+h\right)}^{3}{\left(\frac{\pi }{L}\right)}^{8}f{\left(t\right)}^{3}\sin\left(\frac{\pi }{L}x\right)\cos^{2}\left(\frac{\pi }{L}x\right)\right]+\\ \frac{b_{1}Hh}{2}\left[-\frac{3}{4}b{\left(H+h\right)}^{3}{\left(\frac{\pi }{L}\right)}^{8}f{\left(t\right)}^{3}\sin^{3}\left(\frac{\pi }{L}x\right)-\frac{5}{4}e{\left(H+h\right)}^{5}{\left(\frac{\pi }{L}\right)}^{12}f{\left(t\right)}^{5}\sin^{3}\left(\frac{\pi }{L}x\right)\cos^{2}\left(\frac{\pi }{L}x\right)\right]+\\ \frac{{5b}_{1}Hh}{32}e{\left(H+h\right)}^{5}{\left(\frac{\pi }{L}\right)}^{12}f{\left(t\right)}^{5}\sin^{5}\left(\frac{\pi }{L}x\right)-\frac{b_{1}H^{3}E}{12}{\left(\frac{\pi }{L}\right)}^{4}f\left(t\right)\sin\left(\frac{\pi }{L}x\right)+P{\left(\frac{\pi }{L}\right)}^{2}f\left(t\right)\sin\left(\frac{\pi }{L}x\right)\\ {-\rho b}_{1}H\ddot{f\left(t\right)}\sin\left(\frac{\pi }{L}x\right){+\rho b}_{1}{Hv}^{2}{\left(\frac{\pi }{L}\right)}^{2}f\left(t\right)\sin\left(\frac{\pi }{L}x\right){-2\rho b}_{1}Hv\dot{f\left(t\right)}\cos\left(\frac{\pi }{L}x\right)-c\dot{f\left(t\right)}\sin\left(\frac{\pi }{L}x\right)\\ \;{+f}_{0}\sin\left(\gamma t\right)=0 \end{array}$$

Then, introducing the dimensionless parameters *q = f*/*L*, *τ = tω_n_*, $\omega_{n}=\frac{\sqrt{3}}{6}\sqrt{\frac{{EH}^{2}\pi^{4}}{{\rho L}^{4}}}$, *H*_1_ = *h*/*H*, *H*_2_ = *H*/*L*, *E*_1_ *= a(T* − *T_M_)*/*E*, *E*_2_ *= b*/*E*, *E*_3_ *= e*/*E*. The continuous simply supported beam is discretized by the Galerkin method.

(5) $$\begin{array}{c} \int_{0}^{l}{[-\rho b}_{1}{HL\omega }_{n}{}^{2}\ddot{q}\sin\left(\frac{\pi }{L}x\right)-{2\rho b}_{1}{Hv\omega }_{n}\dot{q}\cos(\frac{\pi }{L}x)-{cL\dot{q}\omega }_{n}\sin\left(\frac{\pi }{L}x\right)-\frac{1}{12}b_{1}{EH}_{2}{}^{3}\pi^{4}q\sin\left(\frac{\pi }{L}x\right)\\ {+E}_{3}\pi^{2}{\left({1+H}_{1}\right)}^{5}q^{5}\sin^{3}\left(\frac{\pi }{L}x\right)\cos^{2}\left(\frac{\pi }{L}x\right)+\frac{5}{32}b_{1}H_{2}{}^{7}H_{1}{EE}_{3}\pi^{12}{\left({1+H}_{1}\right)}^{5}q^{5}\sin^{5}\left(\frac{\pi }{L}x\right)+\\ E_{2}\pi^{8}{\left({1+H}_{1}\right)}^{3}q^{3}\sin\left(\frac{\pi }{L}x\right)\cos^{2}\left(\frac{\pi }{L}x\right)-\frac{5}{8}b_{1}H_{2}{}^{7}H_{1}{EE}_{3}\pi^{12}{\left({1+H}_{1}\right)}^{5}q^{5}\sin^{3}\left(\frac{\pi }{L}x\right)\cos^{2}\left(\frac{\pi }{L}x\right)\\ -\frac{3}{8}b_{1}H_{2}{}^{5}H_{1}{EE}_{2}\pi^{8}{\left({1+H}_{1}\right)}^{3}q^{3}\sin^{3}\left(\frac{\pi }{L}x\right)+\frac{5}{32}b_{1}H_{2}{}^{7}H_{1}{EE}_{3}\pi^{12}{\left({1+H}_{1}\right)}^{5}q^{5}\sin^{5}\left(\frac{\pi }{L}x\right)\\ {+f}_{0}\sin(\gamma t)\sin\left(\frac{\pi }{L}x\right)]=0 \end{array}$$

where:

$$\begin{array}{cc} \int_{0}^{L}\sin^{2}\left(\frac{\pi }{L}x\right)dx=\frac{L}{2} & \int_{0}^{L}\sin\left(\frac{\pi }{L}x\right)\cos\left(\frac{\pi }{L}x\right)dx=0\\ \int_{0}^{L}\sin^{4}\left(\frac{\pi }{L}x\right)dx=\frac{3L}{8} & \int_{0}^{L}\sin^{2}\left(\frac{\pi }{L}x\right)\cos^{2}\left(\frac{\pi }{L}x\right)dx=\frac{L}{8}\\ \int_{0}^{L}\sin^{6}\left(\frac{\pi }{L}x\right)dx=\frac{5L}{16} & \int_{0}^{L}\sin^{4}\left(\frac{\pi }{L}x\right)\cos^{2}\left(\frac{\pi }{L}x\right)dx=\frac{L}{16} \end{array}$$

Next, rearranging the differential equation, the dimensional vibration differential equation is yielded:

(6) $$\ddot{q}+q+c_{0}\dot{q}+k_{1}q+k_{3}q^{3}+k_{5}q^{5}+F_{0}f_{0}\sin(\gamma t)=0$$

where: 

$\begin{array}{c} c_{0}=\sqrt{\frac{12}{{EH}_{2}{}^{4}{\rho b}_{1}{}^{2}\pi^{4}}}c,\;k_{1}{=-6E}_{1}H_{1}\left({1+H}_{1}\right)-\frac{12P}{{6H}_{2}{}^{3}{L\pi }^{2}E}-\frac{{12\rho V}^{2}}{H_{2}{}^{2}\pi^{2}E},\;k_{3}=\frac{9}{8}E_{2}H_{2}{}^{2}\pi^{4}H_{1}{{(1+H}_{1})}^{3}\;\\ k_{5}=-\frac{15}{64}E_{3}H_{2}{}^{4}\pi^{8}H_{1}{{(1+H}_{1})}^{5},\;F_{0}=-\frac{48}{\pi^{5}b_{1}H_{2}{}^{3}E} \end{array}$

## 3. Numerical Example and Parametric Effects

For the axially moving laminated SMA beam, give *L* = 0.5 m, b_1_ = 0.05 m, *H* = 0.02 m, *E* = 206 GPa, *ρ* = 7900 kg/m^3^, and *T* = 300 K. The parametric effects on system steady-state response are analyzed.

### 3.1. Effect of Random Intensity

**Figure 2:**
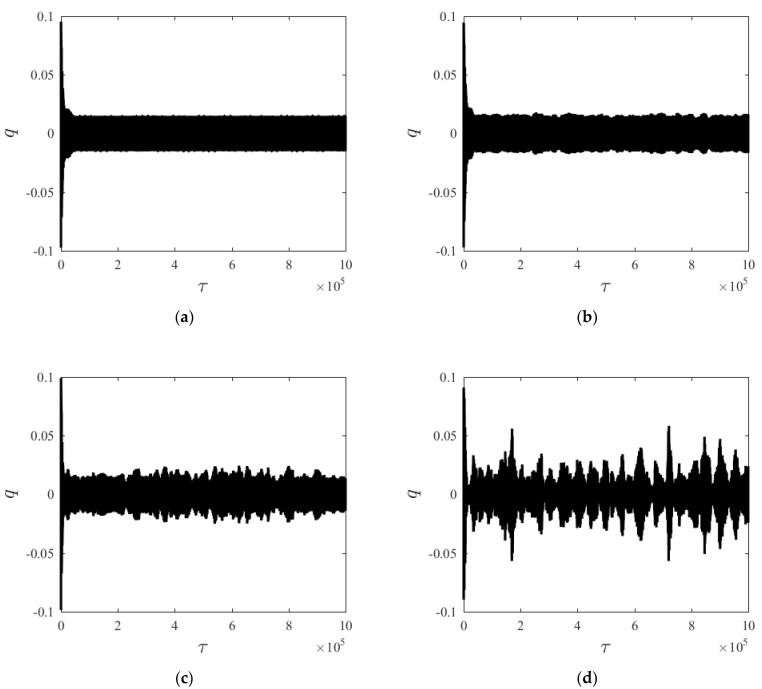
Time histories of *q* under different random intensities. (**a**) D1 = 0, (**b**) D1 = 10, (**c**) D1 = 100, (**d**) D1 = 500.

Figure 2 compares the time histories of the system steady-state response to different random intensities (D), given *v* = 10 m/s, *γ* = 0.97. At D = 0, the system steady-state response consists of a periodic motion, as shown in Figure 2a. As random perturbation intensity increases, transverse vibration amplitude changes randomly near 0.01, and the periodic motion disappears. A similar conclusion was found in Ref. [38] (Figure 7); as the random perturbation intensity increases, the phase diagram changes into a diffused limit cycle. It means that the steady-state response becomes more random and large amplitude vibration appears. Comparison of the four groups of time histories reveals that, with the appearance of random perturbation, a relatively large vibration amplitude appears in the system; meanwhile, the time history curve becomes less orderly.

**Figure 3:**
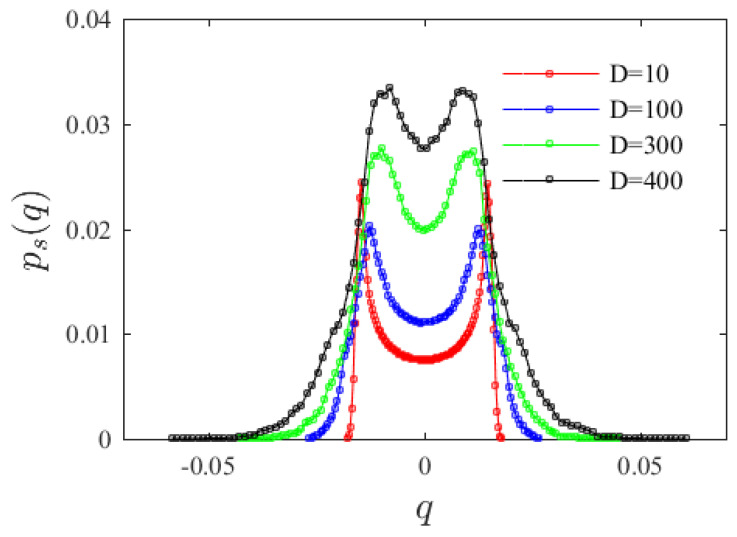
Variation of system marginal probability density function with random perturbation intensity.

Equation (4) is solved, and the system steady-state probability density function is simulated using the Monte Carlo algorithm, which is a method that uses random numbers to solve many computational problems based on probability and statistical theory methods. During the calculation, 10^5^ sample numbers are given by adopting Rung–Kutta fourth-order algorithm, the Monte Carlo simulation is run until the steady state, in the statistical sense, is reached. Figure 3 considers the effects of different random perturbation intensities on the marginal density corresponding to the system steady-state probability density function. It can be seen that increasing random perturbation intensity does not change the peak numbers in the probability density function (PDF) curves, but the value of the peaks gradually flattens and approach the origin. That is, noise intensity will not induce a phase transfer in the system.

### 3.2. Effect of Axial Velocity

**Figure 4:**
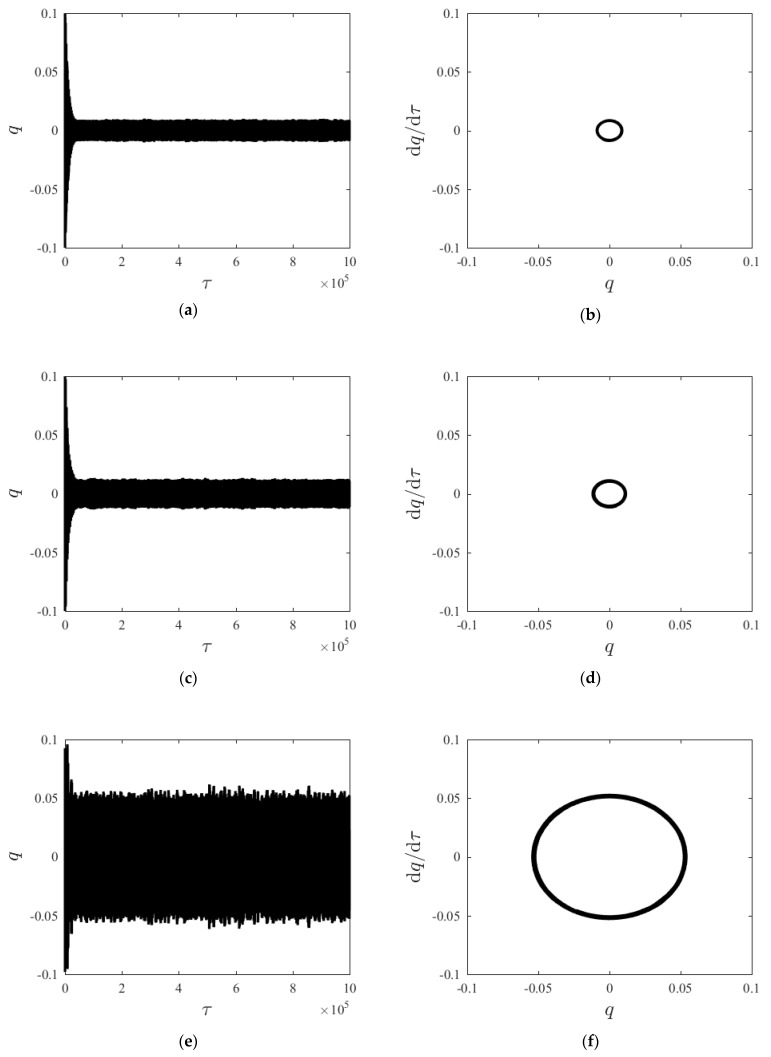

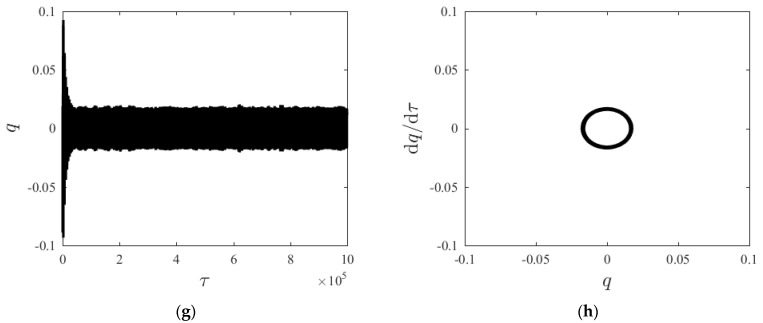
Time history curves and phase diagrams of *q* at different axial velocities *v*. (**a**) displacement–time history curve at *v* = 10 m/s, (**b**) displacement–velocity phase diagram at *v* = 10 m/s, (**c**) displacement–time history curve at *v* = 50 m/s, (**d**) displacement–velocity phase diagram at *v* = 50 m/s, (**e**) displacement–time history curve at *v* = 90 m/s, (**f**) displacement–velocity phase diagram at *v* = 90 m/s, (**g**) displacement–time history curve at *v* = 120 m/s, (**h**) displacement–velocity phase diagram at *v* = 120 m/s.

Given parameters: *γ* = 0.96, *D* = 10. Figure 4 compares the time histories and phase diagrams of the system steady-state response to different axial velocities. As axial velocity increases, the system steady-state response gradually increases from small-amplitude oscillation; however, as axial velocity further increases to a given limit, the steady-state response amplitude gradually decreases. 

**Figure 5:**
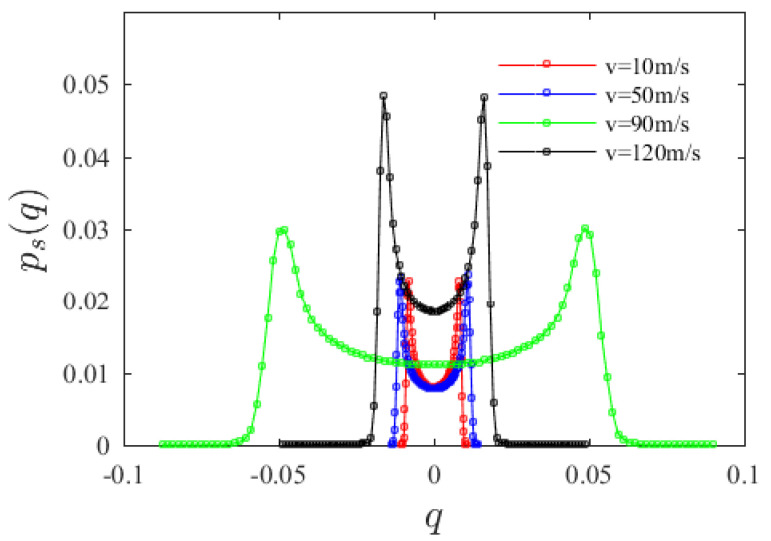
Variation of system marginal probability density function with axial velocity *v*_0_.

From Figure 5, the value of *q* at the marginal probability density peak first increases then decreases with increasing axial velocity. When the axial velocity is less than 90 m/s, with the increase in axial velocity, the peaks’ values in the PDF curves gradually move far away from the origin. The results of the Monte Carlo simulation further show that when the axial velocity is low, the lower the velocity, the smaller the steady-state response amplitude. When the axial velocity is greater than 90 m/s, with the increase in axial velocity, the peaks’ values in the PDF curves become close to the origin.

**Figure 6:**
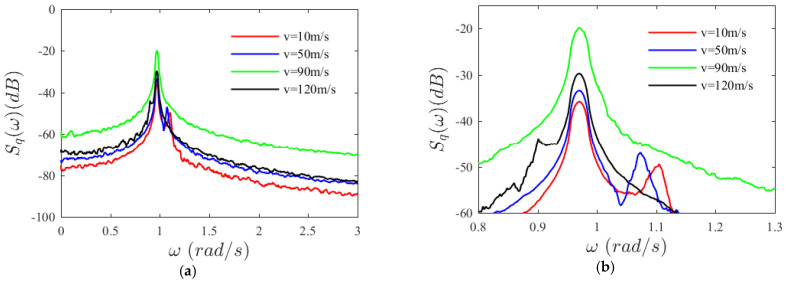
Variation of power spectral density with axial velocity. (**a**) *ω* from 0 to 3 rad/s (**b**) *ω* from 0.8 to 1.3 rad/s.

As axial velocity increases, the shape of the marginal probability density curve does not change qualitatively. Comparison of the power spectral density curves of the transverse displacement of the laminated SMA beam under four different velocities reveals that, under a small velocity, the power spectral density curve contains several energy-concentrated frequency components. The energy at the natural frequency is prominent, whereas those at the subsequent frequency components are less impressive; as axial velocity increases, the second frequency component gradually disappears, as shown in Figure 6. With the increase in axial velocity, the system internal energy shows the following transformation: the width of the spectrum first narrows sharply then gradually broadens, with the maximum standing at the system natural frequency all the time. The power spectral density value at the system natural frequency first increases and then begins to decrease when the velocity is greater than *v* = 90 m/s. 

## 4. Discussion

In this paper, nonlinear random parameter vibration of variable-speed axially moving SMA-laminated beam is studied. Compared with the standard limit cycle oscillation of deterministic systems, the time history curve of SMA-laminated beams shows a large oscillation attributed to the random axially moving velocity disturbance. With the increase in the intensity of random axial velocity disturbance, the randomness of the response time history curve of the system increases. As a result, the time history curve becomes more disordered. 

Laminated structures are seldom considered in the modeling of axially moving beams or plates in the existing literature, most of which studied nonlinear dynamics such as internal resonance, principal resonance, and bifurcation chaos of axially moving beams and plates. The laminated structure studied in this paper is a common structural form consisting of SMA as the surface and linear elastic material as the sandwich. More importantly, the effect of random axial velocity disturbance on nonlinear random parameter vibration of laminated structures in this paper is rarely mentioned in the existing literature. 

Ref. [38] considered the nonlinear random vibration of axially moving SMA-laminated beams under the simultaneous presence of transverse harmonic excitation and random disturbance. Its random excitation is forced excitation in essence. In this paper, the influence of velocity random disturbance is studied, which is parametric random excitation in essence. So, there are essential differences between this paper and Ref. [38]. However, no matter what kind of random factor exists, the time history curve or phase diagram of the system response will be greatly oscillated, and the greater the intensity of the random factor, the stronger the randomness of the system response will be. The conclusion obtained in Figure 7 in Ref. [38] is similar to that in this paper, and this conclusion can be verified mutually.

The SMA Falk’s polynomial model is used to analyze the effects of axial movement and random perturbation in this paper. The advantage of this polynomial model is that it can be used to describe the basic constitutive relations of SMA and it is widely used, while the limitation is that it is simple. In the following work, the nonlinear stochastic dynamics of SMA-laminated plates under the coupling action of multiple fields (including temperature fields) based on complex SMA constitutive relations model will be considered.

## 5. Conclusions

The parametric random vibration of axially moving laminated SMA beams subject to random perturbation was investigated. The dynamic equation of laminated SMA beams subject to uniform transverse loading was established. The random vibration equation was numerically simulated via the Monte Carlo method. The system steady-state response was analyzed in the time and frequency domains: 

As random perturbation intensity increases, large-amplitude oscillation appears randomly in the steady-state response. However, the marginal density curve of the system steady-state response does not change qualitatively. Changed random perturbation intensity does not induce a phase transfer in the system. 

As the axial velocity increases, the system steady-state response gradually increases from small-amplitude oscillation and then gradually decreases. As the axial velocity gradually decreases, the width of the spectrum first narrows sharply then gradually broadens. A second energy-concentrated frequency component appears on the power spectral density curve, with the maximum width standing at the system natural frequency all the time. 

## Data Availability

The data presented in this study are available on request from the corresponding author.
